# Phylogeographic patterns of the yellow fever virus around the metropolitan region of São Paulo, Brazil, 2016–2019

**DOI:** 10.1371/journal.pntd.0010705

**Published:** 2022-09-23

**Authors:** Marielton dos Passos Cunha, Amaro Nunes Duarte-Neto, Shahab Zaki Pour, Bárbara Brito de Souza Pereira, Yeh-Li Ho, Beatriz Perondi, Jaques Sztajnbok, Venancio Avancini Ferreira Alves, Luiz Fernando Ferraz da Silva, Marisa Dolhnikoff, Paulo Hilário Nascimento Saldiva, Paolo Marinho de Andrade Zanotto

**Affiliations:** 1 Laboratory of Molecular Evolution and Bioinformatics, Department of Microbiology, Biomedical Sciences Institute, University of São Paulo, São Paulo, Brazil; 2 Pathology Department, Faculty of Medicine, University of São Paulo, São Paulo, Brazil; 3 Intensive Care Unit, Division of Clinical Infectious and Parasitic Diseases, Clinical Hospital, Faculty of Medicine, University of São Paulo, São Paulo, Brazil; 4 Yellow Fever Crisis Committee, Clinical Hospital, Faculty of Medicine, University of São Paulo, São Paulo, Brazil; 5 Institute of Infectology Emílio Ribas, São Paulo, Brazil; 6 Service of Verification of Deaths of the Capital–University of São Paulo, São Paulo, Brazil; WRAIR, UNITED STATES

## Abstract

From 2016 to 2019, the largest outbreak caused by the Yellow Fever virus (YFV) in the 21^st^ century in the Americas occurred in southeastern Brazil. A sylvatic cycle of transmission was reported near densely populated areas, such as the large metropolitan area of the city of São Paulo. Here, we describe the origin, spread, and movement of the YFV throughout the state of São Paulo. Whole-genome sequences were obtained from tissues of two patients who died due to severe yellow fever, during 2018–2019. Molecular analysis indicated that all analyzed tissues were positive for YFV RNA, with the liver being the organ with the highest amount of viral RNA. Sequence analysis indicates that genomes belonged to the South American genotype I and were grouped in the epidemic clade II, which includes sequences from the states of Goiás, Minas Gerais, and São Paulo of previous years. The analysis of viral dispersion indicates that the outbreak originated in Goiás at the end of 2014 and reached the state of São Paulo through the state of Minas Gerais after 2016. When the virus reached near the urban area, it spread towards both the east and south regions of the state, not establishing an urban transmission cycle in the metropolitan region of São Paulo. The virus that moved towards the east met with YFV coming from the south of the state of Rio de Janeiro, and the YFV that was carried to the south reached the Brazilian states located in the south region of the country.

## Author summary

The Yellow Fever virus (YFV) has caused serious public health issues in Africa and the Americas. YFV infections can vary from asymptomatic to severe clinical conditions, which may culminate in death. In Brazil, the main viral origin of YFV outbreaks is the states located in the Amazon Basin. In the present study, we detected YFV RNA in several tissues, including the heart, lung, brain, kidney, spleen, pancreas, liver, and testicle in two human cases. The YFV characterized in the present study belong to the South American genotype I, clade II, and they are associated with viral spread throughout the state of São Paulo after intense viral circulation in the metropolitan region in 2018. Future investigations are necessary for a better understanding of the complex ecological factors involved in viral dispersion from the Amazon Basin.

## Introduction

The Yellow Fever virus (YFV) is an enveloped arbovirus of the family *Flaviviridae*, genus *Flavivirus*, with a single-stranded, positive-sense RNA virus of approximately 11 kb genome encoding a single polyprotein that is cleaved into three structural, including capsid (C), membrane (M) and envelope (E), and seven non-structural (NS) proteins named NS1, NS2A, NS2B, NS3, NS4A, NS4B, and NS5 [1]. Based on the genetic characteristics, the YFV comprises a single serotype with four genotypes: (*i*) East Africa, (*ii*) West Africa, (*iii*) South American I (SA-I), and (*iv*) South American II (SA-II) [2–4] that may have diverged hundreds of years ago [2,5] following a potential West African origin [2,6]. The infection is naturally initiated by mosquitoes. At the endothelial system, virions are carried by dendritic cells to regional lymph nodes for antigen presentation. This process of onset of infection is followed by the viraemic phase when the virus also migrates to multiple organs. In some cases, a toxaemic phase follows.

YFV is maintained in sylvatic cycles of transmission involving non-human primates (NHP) and sylvatic mosquito species such as *Haemagogus* spp. and *Sabethes* spp. in the Americas, and *Aedes africanus* in Africa. Urban and intermediate cycles of transmission involving humans and *Aedes* spp. have also been reported in Africa [7]. The main urban vector of YFV in Africa, the *Aedes aegypti* mosquito, also transmits Dengue (DENV), Chikungunya (CHIKV), and Zika (ZIKV) viruses to humans in tropical and subtropical areas [8–10]. In both cycles, clinical manifestations range from asymptomatic to fatal conditions [11]. In Brazil, the South American genotype I circulating in a sylvatic environment in the Amazon Basin, including countries such as Brazil, Peru, Bolivia, Colombia, Ecuador, Venezuela, French Guiana, Suriname, and Guyana and is the main group involved in yellow fever epidemics [12,13].

Recently, YFV reemerged in southeast Brazil with intense circulation in the four states of the region, including São Paulo, Rio de Janeiro, Minas Gerais, and Espírito Santo, followed by cases in the northeast and south regions of the country [14–19]. In Brazil, yellow fever (YF) cases are being related to forested environments, and more recently reaching peri-urban areas at the city-forest interface, as the metropolitan region of São Paulo (MRSP) [18,20]. Despite the proximity to urban areas, only sylvatic cycles of transmission have been reported for the last decades in the country [18].

From January to August 17, 2018, 537 fatal cases were reported in the state of São Paulo [21]. Since the beginning of the 21^st^ century, YFV enzootic areas have expanded from the north and central regions to the east coast of Brazil. The advance of yellow fever in the east, which is the most populated region of the country, has led the Ministry of Health to recommend nationwide vaccination for yellow fever [22]. While eradication is not feasible due to the sylvatic cycles of transmission, large-scale vaccination coverage is believed to provide considerable protection against a potential re-urbanization of YFV transmission [23].

Since 2016, an epidemic and several epizootics of yellow fever affecting mainly howler monkey populations, have occurred in regions with low vaccination coverage [18,24]. Here, we described the whole-genome sequencing of two yellow fever cases that occurred in late 2018 and early 2019, in two different locations of the state of São Paulo. The main objective of the present study was to analyze and compare YFV sequences from recent cases to sequences available on GenBank since 2015 (all from clade II of the outbreak) in the states of Goiás, Minas Gerais, and São Paulo, to better estimate the routes of introduction, dispersion, and escape of the virus during the entire period of the outbreak.

## Material and methods

### Ethical statement

The human autopsies reported in this study were performed after obtaining the written consent of the family members and following the protocol approved by the research ethics committee of the Clinical Hospital of the University of São Paulo School of Medicine (HCFMUSP) (CAAE protocol number: 18781813.2.0000.0068). All the methods were performed following the relevant guidelines and regulations of the ethics committee of the HCFMUSP following the approved protocol. All laboratory procedures listed below were performed in a biosafety level 2 laboratory (BSL-2), under the Brazilian standards of the Ministry of Health for Biological Agents Risk Classification [25].

### Patients and samples

From November/2018 to February/2019, we investigated three patients suspected of yellow fever, all of which were later diagnosed as fatal cases of YFV infection. The suspected case definition of yellow fever was established by the Brazilian Ministry of Health in consonance with the Health Department of the state of São Paulo. Suspected yellow fever cases are defined as illness presenting sudden onset high fever associated with jaundice and/or haemorrhage who had lived or had visited areas with evidence of YFV circulation (*i*.*e*., epizootics in NHP or isolation of YFV from vectors), regardless of the vaccine status, during the preceding 15 days.

Confirmed cases had compatible clinical presentation and laboratory confirmation by at least one of the following methods: (*i*) Seropositive by Immunoglobulin M antibody capture enzyme-linked immunosorbent assay (MAC-ELISA); (*ii*) detection of YFV RNA by Real-time quantitative reverse transcription-polymerase chain reaction (RT-qPCR) in blood samples; (*iii*) virus isolation; (*iv*) histopathology compatible with yellow fever hepatitis with detectable antigen in tissues by immunohistochemistry technique. All cases received the definitive laboratory diagnosis of yellow fever by the Adolfo Lutz Institute, the state Reference Laboratory. Previous exposure or co-infection by Hepatitis A virus (HVA), B (HBV), C (HCV), Cytomegalovirus (CMV), Herpes virus (HSV), DENV, CHIKV, Human Immunodeficiency virus type 1 (HIV-1), Leptospira, and other non-infectious diseases aetiologies for acute hepatitis were accessed and cases were excluded following clinical diagnostic methods.

Epidemiological data, including demographic aspects, clinical data, including pre-existing medical conditions, clinical signs and symptoms, and in-hospital follow-up until death, as well as laboratory data, including diagnostic tests, were collected from all patients.

### Autopsy protocol and tissue processing

The Service of Verification of Deaths of the Capital—USP (SVOC-USP) investigated deaths due to yellow fever from November/2018 to February/2019. Autopsies were performed following the *Letulle* technique, where all the organs were removed *en masse* (one block), requiring dissection organ by organ to examine them individually. Briefly, the dissection to all the patients were performed in the following organs: (*i*) heart; (*ii*) lung; (*iii*) brain; (*iv*) kidney; (*v*) spleen; (*vi*) pancreas; (*vii*) liver; and (*viii*) testicle.

### Molecular characterization

Nucleic acid extraction from all collected tissues using 50 mg of the material was performed using the TRIzol reagent (Life Technologies, Carlsbad, CA, USA) and carried out according to the manufacturer’s instructions. Molecular detection of YFV was performed by AgPath-ID One-Step RT-PCR Reagents (Ambion, Austin, TX, USA) with specific primers/probe, as previously described [26]. To identify cases of rare adverse events from yellow fever vaccination we used specific primers/probe for the detection of the attenuated viral strain 17D, the vaccine virus [27]. To quantify the genomic viral concentration in each tissue, we used a double-stranded DNA fragment containing the amplification region (Exxtend, SP, Brazil), which from several known molecules, was diluted and used to estimate the viral RNA concentration by linear regression. RT-qPCR reactions were run in ABI7500 equipment (Thermo Fisher Scientific, Waltham, MA, USA). To visualize the spatial location of the positive patients, we used R software, geobr package, (MIT license https://ipeagit.github.io/geobr/) [28] to create the maps.

### Sequencing and viral genome assembly

Based on the RNA viral concentration and in our previous protocol to obtain viral complete genomes from human tissues [18,29], positive samples had the total RNA re-extracted by the abovementioned procedure. RNA samples were then purified and concentrated with DNase I and RNA Clean and Concentrator -5 kit (Zymo Research, Irvine, CA, USA). The paired-end RNA libraries were constructed and validated using the TruSeq Stranded Total RNA HT sample prep kit (Illumina, San Diego, CA, USA). Sequencing was done at the Core Facility for Scientific Research–University of São Paulo (CEFAP-USP/GENIAL) using the Illumina MiSeq platform. Each sample was barcoded individually, which allowed the separation of reads for each patient. Short unpaired reads and low-quality bases and reads were removed using Trimmomatic version 0.39 (LEADING:20 TRAILING:20 SLIDINGWINDOW:4:25 MINLEN:36) [30]. Consensus genomes were assembled with paired-end reads using Bowtie2 version 2.4.1 using default parameters [31]. To access the coverage and generate the consensus genome (Fig A in S1 Text), we used UGENE v.33.0 [32].

### Data sets

All the complete genomic sequences available for YFV (until August 20, 2020), and their associated information, such as location and year of detection were recovered from the National Center for Biotechnology Information (NCBI) (https://www.ncbi.nlm.nih.gov/genbank/) website in GenBank format. All sequences were converted into FASTA format, and to avoid duplicated and low-quality sequences with many unknown nucleotides (N), we set an exclusion threshold for sequences that had more than 1% of “N” concerning their total length (https://biopython.org/wiki/Sequence_Cleaner). These sequences were grouped with the sequences isolated from patients in São Paulo 2018–2019, the FASTA sequences were aligned using Clustal Omega [33] and recombinant sequences were screened using all algorithms implemented in RDP5 Beta 5.05 program (RDP, GENECONV, MaxChi, BootScan, and Siscan) using the standard settings [34]. The alignment of recombinant free sequences was manually inspected and edited using the program AliView v.1.18 [35].

To determine which genotypes and lineages circulated in the state of São Paulo during the outbreak, two datasets were constructed: (*i*) the first (dataset-1) included all the sequences deposited at GenBank (before August—2020), with the sequences determined in our study (n = 264 sequences) (Table A in S1 Text); (*ii*) the second (dataset-2), based in the phylogenetic analysis of the dataset-1, included all sequences deposited at GenBank (before August—2020) belonging to the genotype SA-I, clade II of the outbreak (sequences isolated from 2015 in the states of Goiás, Minas Gerais, and São Paulo states) (n = 91 sequences) (Table B in S1 Text), according to our previous publication [18].

### Phylogenetic analysis

Phylogenetic inference was performed with all the curated datasets. The phylogenetic tree was reconstructed based on the full-length, curated coding sequences using the Maximum Likelihood (ML) method implemented in IQ-TREE 1.5.5 [36]. The robustness of the groupings observed was assessed using an ultrafast bootstrap approximation (UFboot) during 1,000 replicates. The ML tree was visualized and plotted using FigTree v.1.4.3 [37]. Taxon labels for sequences used in this work had the format: genotype/accession number/local of isolation/date of isolation.

### Phylogeographic analysis

All the sequences characterized as clade II were used to reconstruct the viral origin and to trace the movement of the C-II sequences (dataset-2). We explored the temporal signal (*i*.*e*., molecular clock structure) and the quality of our data set using TempEst v.1.5.3 [38]. The spatiotemporal spread was reconstructed under a Bayesian framework implemented in BEAST v.1.10.4 [39], using the general time-reversible model incorporating invariant sites with gamma-distributed rate variation substitution model (GTR+I+G), as described by the Bayesian information criterion (BIC) using jModelTest 2.1.10 [40]. Based on previous estimates of evolutionary dynamics of related YFV [17,41], we tested uncorrelated relaxed molecular clocks assuming a log-normal distribution, in combination with non-parametric population growth models: (*i*) the standard Bayesian skyline plot (BSP; 10 groups) [42], (*ii*) the Bayesian skyride plot [43], and (*iii*) the Bayesian skygrid model [42] (Tables C and D in S1 Text). Phylogeography patterns and parameters were estimated by running a Markov Chain Monte Carlo (MCMC) for 200 million states and sampling every 200.000 states with 10% burn-in. The effective sample size (ESS) and convergence were examined with Tracer v.1.7.1 [44]. The maximum clade credibility (MCC) tree was also visualized with FigTree v.1.4.3 [37]. To calculate the log marginal likelihood for demographic model selection, we used the path sampling (PS) [45,46] and the stepping-stone (SS) sampling [47] approaches by running 100 path steps of 1 million iterations each. The spatiotemporal spread was visualised with spreaD3 v.0.9.7.1 [48].

### Data sequences

The new sequences here characterized were deposited in GenBank under the accession numbers MW308134 and MW308135.

## Results

### YFV epidemiological surveillance in São Paulo, 2016–2019

**Fig 1:**
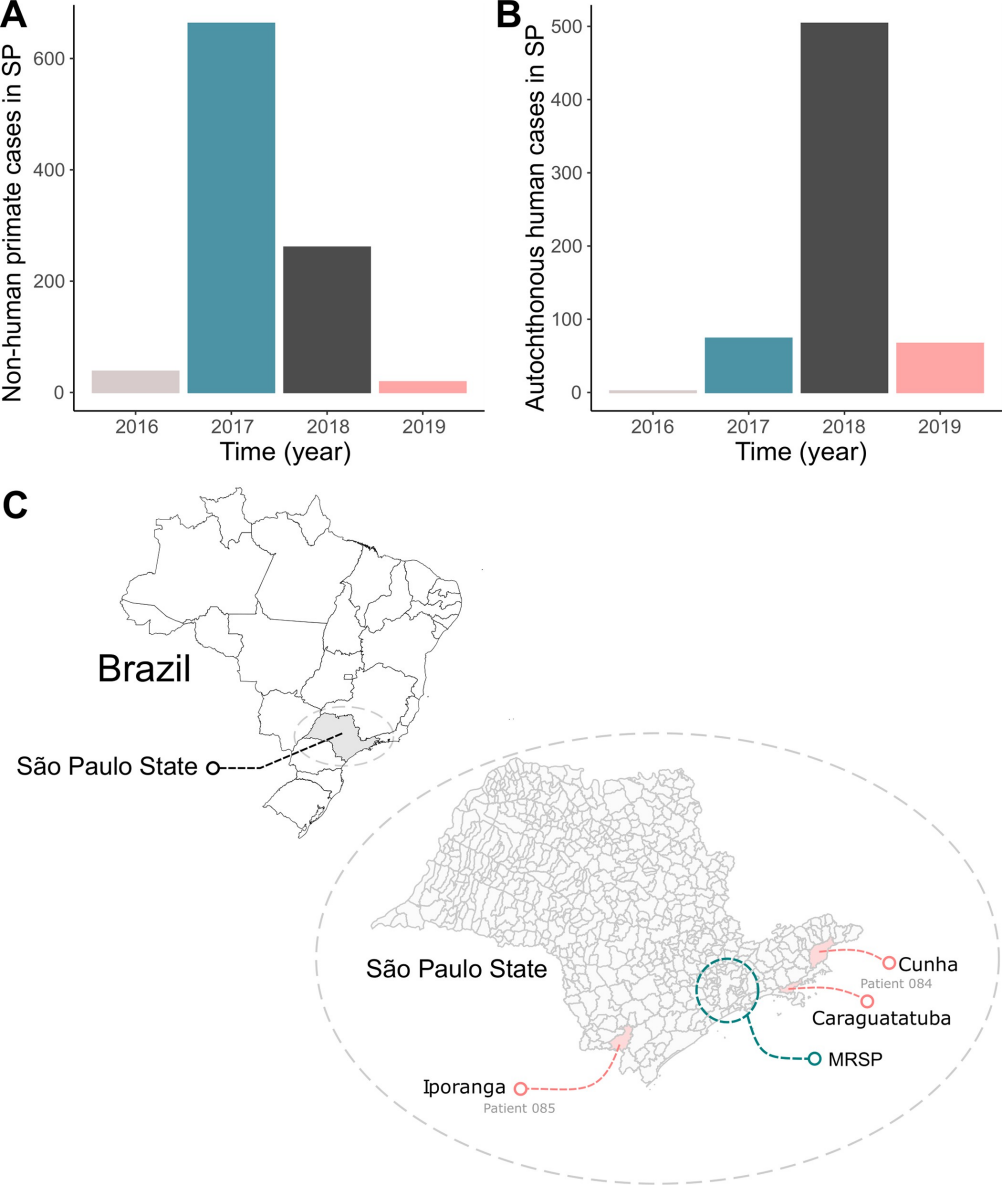
Epidemiological situation of the YFV outbreak in São Paulo, 2016–2019. (**A**) The number of YFV positive NHPs in São Paulo, from 2016–2019. (**B**) The number of YFV-positive human cases in São Paulo, from 2016–2019. (**C**) Location of the two fatal human cases studied in the transition from 2018 to 2019. We used R software, geobr package, (MIT license https://ipeagit.github.io/geobr/) to create the maps produced in this Fig to visualize the spatial location.

YFV emerged in the state of São Paulo and was first recognized in early 2016, through the detection of two human cases. The monitoring of epizootics revealed an ongoing occurrence of yellow fever in NHP. In 2016 and 2017, the number of NHP cases reported was higher than in humans. Since the end of 2017, YFV was reported in an extensive area where there was no recommendation for vaccination, mainly in the MRSP. In late 2018, a specific case was observed in the Atlantic Forest in the eastern region of the state, near the border with the state of Rio de Janeiro. In early 2019, some cases were observed in the south of the state, close to the border with the state of Paraná, and in the last two years of the epidemic (2018 and 2019), the number of human cases has suppressed the number of reported NHP cases (Fig 1A–1C).

From October 2018 to February 2019, three patients suspected of fatal yellow fever entered the SVOC-USP in the city of São Paulo. All patients had organs that tested positive for RT-qPCR. Two patients (patient 084 and patient 085) were confirmed positive for sylvatic YFV, and one of them tested positive for the vaccine strain YFV-17DD.

### Clinical and demographic features associated with the sylvatic YF cases

The first case (patient 084) was a 26-year-old man, from the city of Cunha, a peasant, without any medical condition. He travelled one week before symptoms onset to Caraguatatuba, a region of Atlantic Forest, to work on a farm. He had no YF vaccination. His disease started on October 20^th^, 2018, presenting asthenia, generalized myalgia, intense headache, fever, oliguria, nausea, vomits, abdominal pain, and developed icterus in the following days. He was transferred to São Paulo to the intensive care unit of the Infectology Institute “Emílio Ribas”, on the 7^th^ day. The initial clinical hypothesis was: YF, leptospirosis, dengue, or Brazilian spotted fever. At admission, he was in a severe clinical condition, with torpor, bradycardia, and uremic, and was put under mechanical ventilation. During hospitalization fulminant hepatitis was diagnosed, with gastrointestinal haemorrhages, convulsions, and acute kidney injury, needing dialysis and multiple hemocomponents transfusion. He died on the 9^th^ day of disease onset. The diagnosis of YF was done by a positive anti-YFV IgM in serum (collected on the 7^th^ day); detectable YFV RNA in the blood by RT-PCR (7^th^ day), and autopsy findings. Based on the demographic history that preceded this patient’s fatal condition, we decided to consider the probable site of infection as Caraguatatuba in the viral spread reconstruction.

The second case (patient 085) was a 58-year-old male, peasant, resident of Iporanga, close to areas with confirmed cases of YF epizootics and other human cases. He had a medical history of high blood pressure and was treated for prostate cancer. His symptoms started on January 15^th^, 2019, with intense headache, fever, malaise, nausea, vomiting, and jaundice. The patient was transferred to IIER ICU with clinical suspicion of yellow fever. He evolved with fulminant hepatitis with coma and multiple organ dysfunction, treated with mechanical ventilation, hemocomponents transfusion, antibiotics, and other intensive measures. The patient died on the 5^th^ day of illness. The diagnosis of YF was done by a positive anti-YFV IgM in serum (collected on the 5th day); detectable YFV RNA in the blood by RT-PCR (4th day), and autopsy findings.

**Fig 2:**
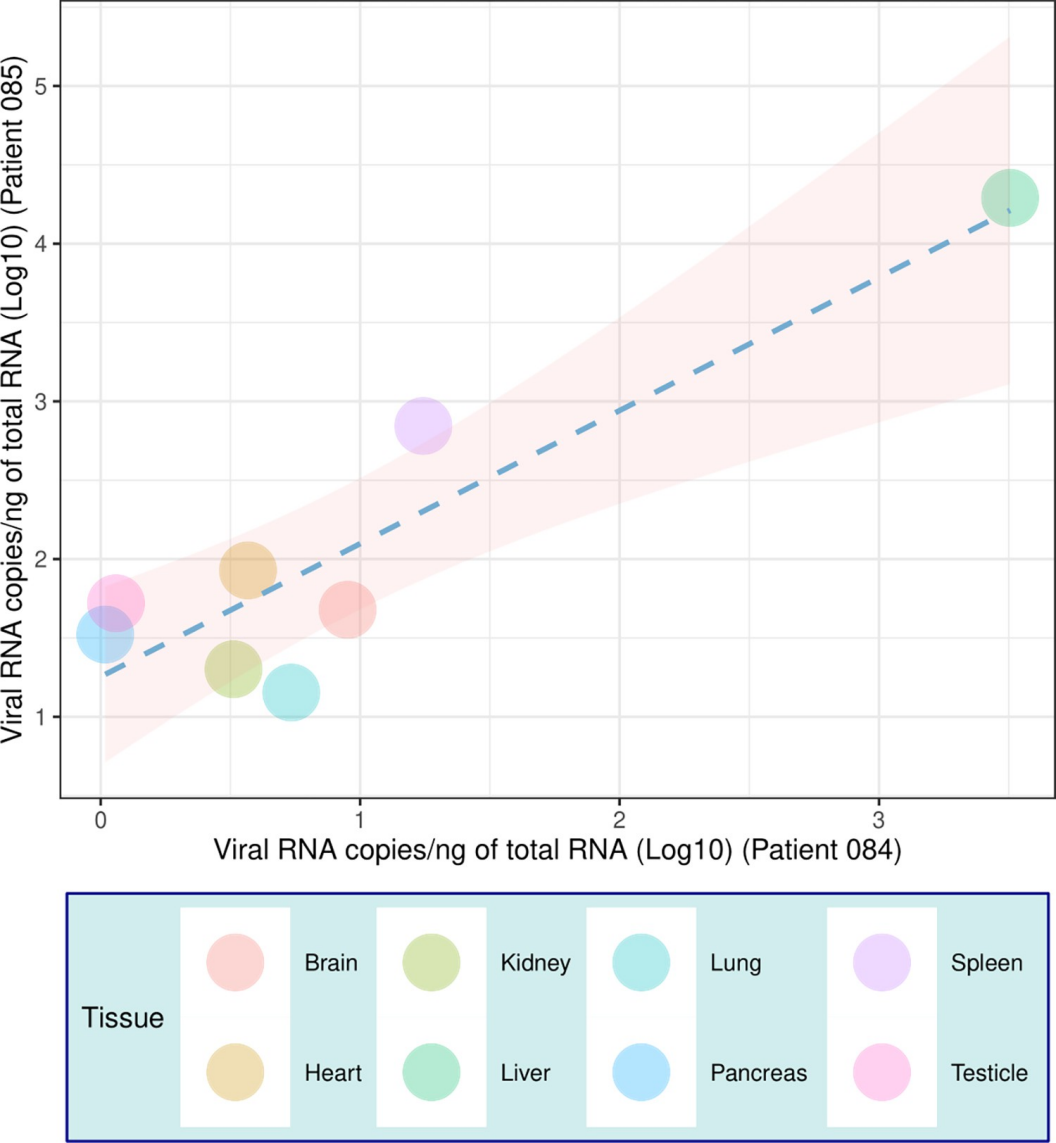
Molecular characterization of the sequenced yellow fever cases, showing the number of YFV RNA copies according to each of the 8 tissues analyzed for positive YFV-sylvatic patients. The different colors represent the different tissues analyzed. The results indicate that both patients present a positive correlation for the quantification of genetic material.

Both cases had mid-zonal hepatitis, with steatotic and apoptotic hepatocytes, sinusoidal congestion and Kupffer cells hyperplasia; diffuse gastrointestinal haemorrhages; pulmonary haemorrhages, lymphoid hypoplasia, and acute tubular necrosis. Liver tissues presented high levels of viral RNA when compared to other tissues (Fig 2).

### Phylogenetic analysis

Based on our previous work that showed the efficiency in recovering quality viral YFV sequences from liver autopsy material of patients with clinical outcomes of death [18], we submitted the total RNA obtained from the patients’ liver to total genomic RNA sequencing. In both, quality sequences were obtained, one with the complete coding region, and the other almost complete, having 20 contiguous bp that were not sequenced, that was assumed as “N” from the 6315–6334 site of the coding sequence (Fig A in S1 Text). However, we chose to keep this sequence in our analysis, since no nucleotide substitution in that region was observed in these positions in all clade II sequences (n = 91 sequences), which were assumed as possibly non-informative sites.

### Phylogenetic characterization

**Fig 3:**
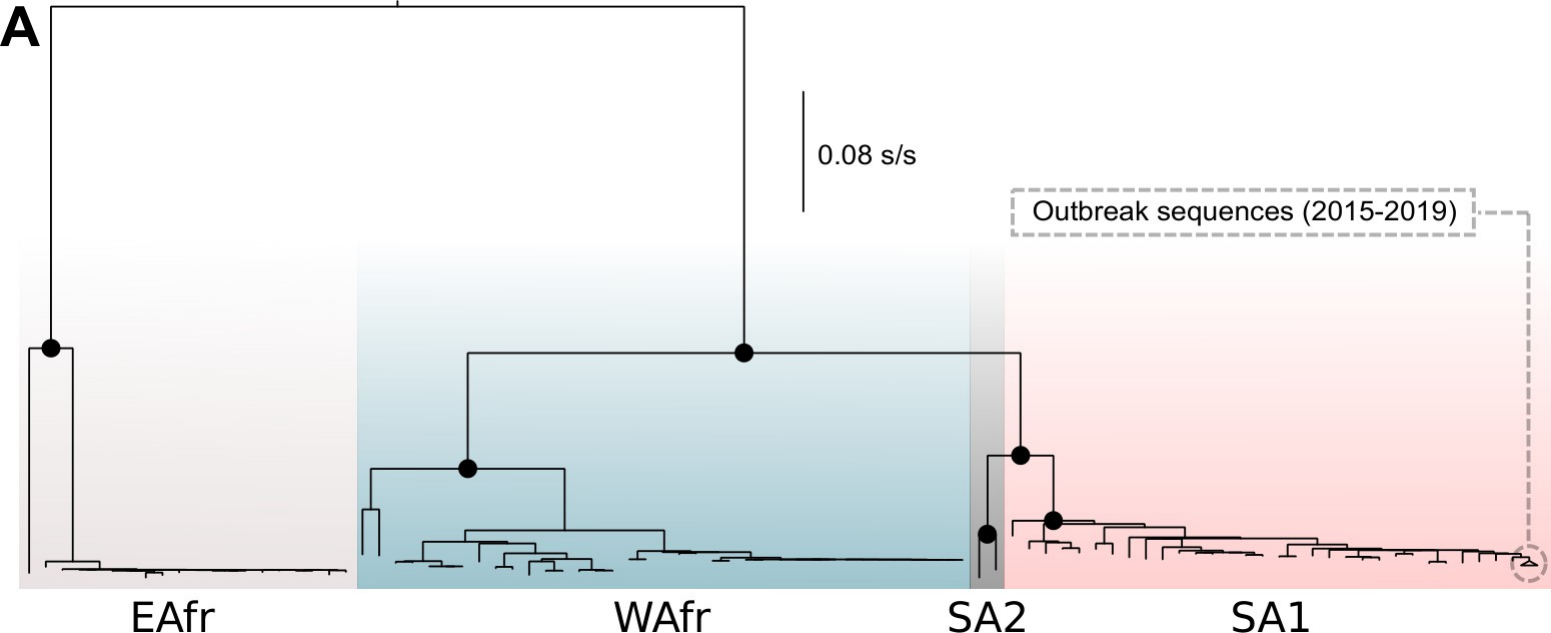
Maximum likelihood phylogenetic trees for YFV based on full-length polyprotein sequences (n = 264) indicate that the sequences associated with the outbreak in Brazil between 2016–2019 grouped within the genotype South American I. The tree is midpoint-rooted and the black circles on the main nodes represent bootstrap support values greater than 0.7. The distinct colors represent distinct genotypes: (*i*) EAfr—East African; (*ii*) WAfr—West African; (*iii*) SA1—South American I; (*iv*) SA2—South American II.

**Fig 4:**
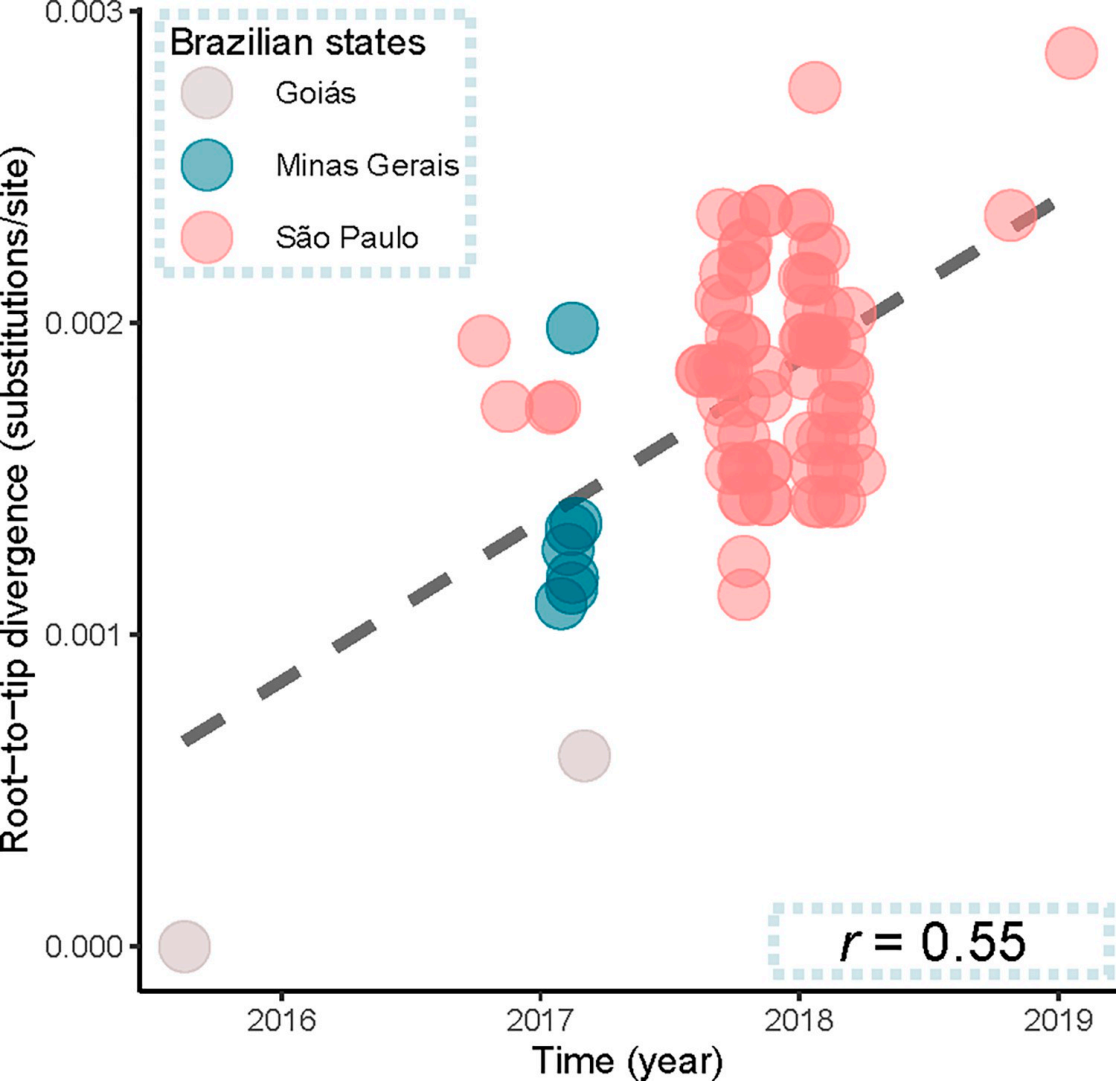
Correlation between genetic divergence and sampling time was obtained by a root-to-tips analysis using the YFV sequences. Root-to-tips distances were inferred using a maximum likelihood phylogeny for the genotype South American I, Clade II, (dataset-2).

The phylogenetic characterization of the two viruses sequenced here, compared to sequences previously characterized and deposited in the GenBank indicated that both sequences grouped within the South American genotype I, within the group of sequences associated with the outbreak identified in Brazil between 2016–2019, including sequences of midwest, southeast and northeast Brazil (Fig 3). The coding region of the two genomes sequenced here was grouped into a multi-Fasta file with another dataset of 89 non-redundant genomes recovered from GenBank (n = 91 sequences), previously filtered to exclude incomplete genomes, sequences with low quality, and sequences without information on location and date of collection. This alignment, with 91 sequences, was generated producing a file with 10.236 nucleotide sites, with 10.073 constant and invariant (constant or ambiguous constant) sites (98.41% of all sites). Among the variant sites, 60 sites were considered informative under parsimony and 202 sites had distinct site patterns. A root-to-tips analysis showed that the virus accumulated genetic diversity over time (*r* = 0.55) (Fig 4).

### Phylogeographic characterization

**Fig 5:**
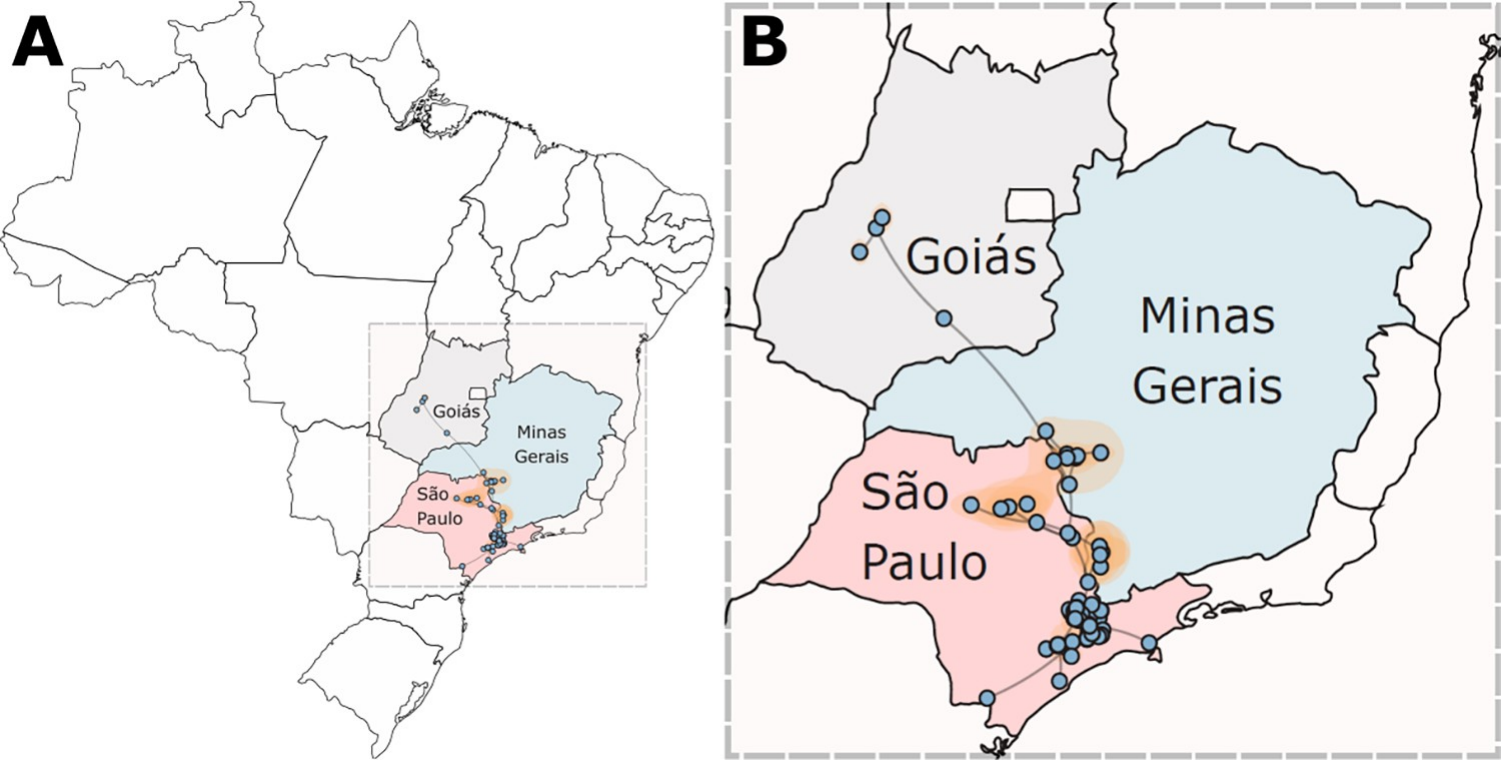
Highest posterior probability migration paths for the YFV Clade II from 2015 to 2019 towards and beyond the MRSP, based on the analysis of 91 complete genomes (dataset 2). (**A**) Migration on the full map of Brazil, and (**B**) migration being viewed up close. The spatiotemporal spread was visualized with spreaD3 v.0.9.7.1, using a GeoJSON file, (MIT license https://www.kaggle.com/datasets/moiseslima/majson?resource=download) to visualize the spatial YFV spread.

The comparison between some of the different models of viral propagation and diffusion (Tables C and D in S1 Text) allowed us to estimate the pattern of viral dispersion with greater accuracy. According to the inferential results (Fig 5), the outbreak began in the state of Goiás in December/2014 (95% HPD = February/2014 –August/2015), and subsequently reached the state of Minas Gerais in September/2015 (95% HPD = November/2014 –May/2016), also reaching the state of São Paulo. In São Paulo, the virus was introduced a single time in February/2016 (95% HPD = September/2015 –July/2016), and since then it has spread to several locations throughout the state reaching back to the state of Minas Gerais after 2016.

## Discussion

Since the last urban cases of yellow fever reported in the Americas in the early 1950s, YFV has been maintained in forested areas through sylvatic cycles of transmission. The largest yellow fever outbreak in the 21^st^ century in the Americas began in 2016 passing through all states of southeast Brazil reaching the south and northeast regions of the country [14,18,19,49]. To reach the state of Minas Gerais, the virus possibly was carried across the state of Goiás, located in the west-central region, as the bridge from its natural maintenance area, the Amazon basin. Goiás is located in a dry savannah area (cerrado), which is a biome physically linked to the Amazon and Atlantic Forest [49,50]. In recommended areas for YF vaccination, the virus circulated silently causing epizootics in NHP. In areas with low vaccination coverage, YFV affected human populations causing a high number of deaths from 2017 to 2018 [18,22].

In the MRSP, the virus began its circulation in 2017, reaching the region from the south Minas Gerais, moving slowly and causing infections in NHP and humans along with its spread [18,24]. Despite circulating in forested regions close to densely inhabited areas in the MRSP during 2017–2018, YFV only infected susceptible people who had reported exposure to forested environments [15,16,18]. In the following epidemiological period (2018–2019), no cases were reported in NHP or humans in the MRSP. This can be explained by two factors: (*i*) YFV collapsed many populations of NHP in the region and (*ii*) the human population adhered to mass vaccination during the outbreak. During 2018–2019, the YF cases were reported mainly in the Vale do Ribeira (south), but also in the east of the state of São Paulo. In the case of the patient who lived in the city of Cunha, he visited a neighboring city (Caraguatatuba) at a time compatible with the viral incubation period, which is about 3 to 6 days before the onset of symptoms [51,52], which is located in the same region in a distance of 76 km away.

Multiple organs were positive for the presence of YFV RNA, as we have shown recently for other cases [15,16,18]. The liver was the organ with a high viral load in both patients. These findings reinforce the well-established evidence that the liver tissue is a replication site of YFV and the notion of multiple tissue compartmentalization in severe cases of infection. In some severe and non-fatal cases, hepatic tissue infection is seen months after the clinical outcome of the disease, causing late hepatitis, with positive molecular detection and evidence of lesions in pathological analyses [53].

In experimental models using NHP, the genome and viral antigens are found in multiple organs with a greater amount of virus, when compared to serum, between one to 30 days post-infection. Interestingly, neutralizing antibodies are found after the seventh day of infection, in consonance with the reduction of viremia and subsequently reduction of virus levels in tissues [54]. Our findings shown in Fig 2 support a model for YFV infection in humans based on autopsy and molecular findings, characterized by hepatitis, nephropathy, coagulopathy, and haemorrhagic vasculopathy that can lead to a fatal condition [16].

The viral sequences recovered from both patients reported here are grouped within the South American genotype I, alongside sequences previously characterized in previous years in São Paulo and neighboring states [14,18,49]. Within the group of outbreak sequences, they were grouped within clade II, which are sequences corresponding to viral isolates that circulated in the states of São Paulo, Minas Gerais, and Goiás [18,49].

The pattern of viral spread observed in the transition from 2018 to 2019 suggested that the densely urbanized area of the MRSP acted as a barrier to viral dispersion, deflecting the virus to spread to the east and south of the state. When comparing the transmission network of the 2015–2019 outbreak with other well-noted outbreaks from the past, we noticed that a very similar pattern of viral movement was observed in Brazil during outbreaks between 1932 and 1942 [50,55,56]. During those outbreaks, YFV reached the state of São Paulo from the Amazon Basin, also through the state of Goiás. Possibly, the maintenance of the viral spread pattern is associated with biological variables associated with virus-host interaction, including vectors and NHP, and geo-environmental variables, such as altitude, annual rainfall, and temperature [57]. As occurred between 1932–1942, YFV reached the MRSP and spread to the east and south regions of the state. Two possible hypotheses can explain the recent virus siege of the MRSP: (*i*) when the first cases of YFV were identified in NHP and human populations close to urban areas in the MRSP, the Department of Health of the state of São Paulo started mass campaigns for vaccination in peri-urban regions, causing an immunological protection belt around the densely populated area of the MRSP, and (*ii*) since the first half of the 20^th^ century, outbreaks in urban environments in Brazil caused by YFV are not documented, and one of the hypothesis is the lack of vector competence of the current *Aedes aegypti* populations in Brazil. However, a recent study demonstrated that different populations of *Aedes aegypti* in Brazil are competent for urban transmission of YFV [58]. Thus, vaccination associated with the circulation of other closely related flaviviruses in urban areas, as well as other ecological and epidemiological factors is believed to be involved in the continuous absence of urban cycles of transmission of YFV in the Americas.

In 2019, the YFV circulation decreased considerably compared to previous years, but with the circulation of the virus associated with clade I in the state of Rio de Janeiro with few cases [59], and in São Paulo associated with the clade II, some cases were reported. After 2019, NHP and human cases of YFV were reported in south Brazil, including the states of Paraná, Santa Catarina, and the Rio Grande do Sul [60–62]. Considering the limited number of YFV sequences available for better evaluation of the movement of YFV in these states, our analysis suggests that YFV that arrived in the state of Paraná moved to the southern region of the state of São Paulo, as they are border states.

## Supporting information

S1 Text **Fig A in S1 Text**. Combined coverage (normalized by the sample average) along the two sequenced Yellow Fever virus (YFV) genomes generated in this study. The genomic position reflects the genomic organization of the YFV, which is organized as follows: a single polyprotein cleaved into three structural, including capsid (C), membrane (M), and envelope (E), and seven non-structural (NS) proteins named NS1, NS2A, NS2B, NS3, NS4A, NS4B, and NS5. The polyprotein is flanked by the 5’ and 3’ ends, which are non-coding. **Table A in S1 Text**. Complete YFV polyprotein sequences used in the phylogenetic analysis (dataset-1) (n = 264). **Table B in S1 Text**. Complete YFV polyprotein sequences used in the phylogeographic analysis (dataset-2) (n = 91). **Table C in S1 Text**. Model comparison of the relaxed molecular clock and demographic growth models through path sampling (PS) and stepping stone (SS) methods. Bold numbers indicate the best fitting model. **Table D in S1 Text**. Comparison among continuous diffusion models for Brazilian sequences.
(DOCX)

## References

[1] Chambers TJ, Hahn CS, Galler R, Rice CM. Flavivirus Genome Organization, Expression, and Replication. Annu Rev. 1990;44: 649–688. 10.1146/annurev.mi.44.100190.003245 2174669

[2] Bryant JE, Holmes EC, Barrett ADT. Out of Africa: A Molecular Perspective on the Introduction of Yellow Fever Virus into the Americas. PLoS Pathog. 2007;3: e75. doi: 10.1371/journal.ppat.0030075 17511518 PMC1868956

[3] Monath TP, Vasconcelos PFC. Yellow fever. J Clin Virol. Elsevier B.V.; 2015;64: 160–173. doi: 10.1016/j.jcv.2014.08.030 25453327

[4] Barrett ADT, Higgs S. Yellow Fever: A Disease that Has Yet to be Conquered. Annu Rev Entomol. 2007;52: 209–229. doi: 10.1146/annurev.ento.52.110405.091454 16913829

[5] Zanotto PM de A, Gould EA, Gao GF, Harveyt PH, Holmestt EC. Population dynamics of flaviviruses revealed by molecular phylogenies. Proc Natl Acad Sci. 1996;93: 548–553. doi: 10.1073/pnas.93.2.548 8570593 PMC40088

[6] Nunes MRT, Palacios G, Cardoso JF, Martins LC, Sousa-Junior EC, Lima CPS de, et al. Genomic and phylogenetic characterization of Brazilian Yellow Fever virus strains. J Virol. 2012; 1–33. doi: 10.1128/JVI.00565-12 23015713 PMC3503022

[7] Abreu FVS de, Ribeiro IP, Ferreira-de-Brito A, Santos AAC dos, Miranda RM de, Bonelly I de S, et al. *Haemagogus leucocelaenus* and *Haemagogus janthinomys* are the primary vectors in the major yellow fever outbreak in Brazil, 2016–2018. Emerg Microbes Infect. Taylor & Francis; 2019;8: 218–231. doi: 10.1080/22221751.2019.1568180 30866775 PMC6455131

[8] Salazar MI, Richardson JH, Sánchez-Vargas I, Olson KE, Beaty BJ. Dengue virus type 2: replication and tropisms in orally infected Aedes aegypti mosquitoes. BMC Microbiol. 2007;7: 9. doi: 10.1186/1471-2180-7-9 17263893 PMC1797809

[9] Costa-da-Silva AL, Ioshino RS, Petersen V, Lima AF, Cunha MDP, Wiley MR, et al. First report of naturally infected Aedes aegypti with chikungunya virus genotype ECSA in the Americas. PLoS Negl Trop Dis. 2017;11. doi: 10.1371/journal.pntd.0005630 28614394 PMC5470658

[10] Romo H, Kenney JL, Blitvich BJ, Brault AC. Restriction of Zika virus infection and transmission in Aedes aegypti mediated by an insect-specific flavivirus. Emerg Microbes Infect. Springer US; 2018;7: 1–13. doi: 10.1038/s41426-018-0180-4 30429457 PMC6235874

[11] Beasley DWC, McAuley AJ, Bente DA. Yellow fever virus: Genetic and phenotypic diversity and implications for detection, prevention and therapy. Antiviral Res. Elsevier B.V.; 2015;115: 48–70. doi: 10.1016/j.antiviral.2014.12.010 25545072

[12] Fernandes NCCA, Guerra JM, Díaz-Delgado J, Cunha MS, Saad L del C, Iglezias SD, et al. Differential yellow fever susceptibility in new world nonhuman primates, comparison with humans, and implications for surveillance. Emerg Infect Dis. Centers for Disease Control and Prevention (CDC); 2021;27: 47–56. doi: 10.3201/eid2701.191220 33350931 PMC7774563

[13] Vasconcelos PFC, Bryant JE, Travassos APA, Tesh RB, Rodrigues SG, Barrett ADT. Genetic Divergence and Dispersal of Yellow Fever Virus, Brazil. 2004;10. doi: 10.3201/eid1009.040197 15498159 PMC3320275

[14] Barbosa CM, Paola N Di, Cunha MP, Rodrigues-jesus MJ, Araujo DB, Silveira VB, et al. Yellow Fever Virus RNA in Urine and Semen of Convalescent Patient, Brazil. Emerg Infect Dis. 2018;24: 176–178. 10.3201/eid2401.171310 PMC5749440 29058663

[15] Duarte-neto AN, Monteiro RA de A, Johnsson J, Cunha M dos P, Pour SZ, Saraiva AC, et al. Ultrasound-guided minimally invasive autopsy as a tool for rapid post-mortem diagnosis in the 2018 São Paulo yellow fever epidemic: Correlation with conventional autopsy. PLoS Negl Trop Dis. 2019;13: e0007625. 31329590 10.1371/journal.pntd.0007625 PMC6675127

[16] Duarte-Neto AN, Cunha M dos P, Marcilio I, Song ATW, de Martino RB, Ho Y, et al. Yellow Fever and Orthotopic Liver Transplantation: new insights from the autopsy room for an old but reemerging disease. Histopathology. 2019; 1–11. doi: 10.1111/his.13904 31087672

[17] Faria NR, Kraemer MUG, Hill S, Jesus JG de, Aguiar RS de, Iani FCM, et al. Genomic and epidemiological monitoring of yellow fever virus transmission potential. Science (80-). 2018;7115: 1–12. doi: 10.1126/science.aat7115 30139911 PMC6874500

[18] Cunha M dos P, Duarte-Neto AN, Pour SZ, Ortiz-Baez AS, Černý J, Pereira BB de S, et al. Origin of the São Paulo Yellow Fever epidemic of 2017–2018 revealed through molecular epidemiological analysis of fatal cases. Sci Rep. 2019;9: 1–10. doi: 10.1038/s41598-019-56650-1 31892699 PMC6938505

[19] Goes de Jesus J, Gräf T, Giovanetti M, Mares-Guia MA, Xavier J, Lima Maia M, et al. Yellow fever transmission in non-human primates, Bahia, Northeastern Brazil. PLoS Negl Trop Dis. 2020;14: e0008405. doi: 10.1371/journal.pntd.0008405 32780745 PMC7418952

[20] Lacerda AB, del Castillo Saad L, Ikefuti PV, Pinter A, Chiaravalloti-Neto F. Diffusion of sylvatic yellow fever in the state of São Paulo, Brazil. Sci Reports 2021 111. Nature Publishing Group; 2021;11: 1–11. doi: 10.1038/s41598-021-95539-w 34381111 PMC8358007

[21] Ministério da Saúde. Boletim epidemiológico—Febre Amarela—17/08/2018. Bol Epidemiológico. 2018; 1–10. Available: http://portalms.saude.gov.br/saude-de-a-z/febre-maculosa/situacao-epidemiologica

[22] Romano APM, Costa ZGA, Ramos DG, Andrade MA, de Jayme VS, de Almeida MAB, et al. Yellow Fever Outbreaks in Unvaccinated Populations, Brazil, 2008–2009. PLoS Negl Trop Dis. 2014;8: e2740. doi: 10.1371/journal.pntd.0002740 24625634 PMC3953027

[23] Shearer FM, Longbottom J, Browne AJ, Pigott DM, Brady OJ, Kraemer MUG, et al. Existing and potential infection risk zones of yellow fever worldwide: a modelling analysis. Lancet Glob Heal. 2018;6: e270–e278. doi: 10.1016/S2214-109X(18)30024-X 29398634 PMC5809716

[24] Hill SC, Souza R, Thézé J, Claro I, Aguiar RS, Abade L, et al. Genomic surveillance of yellow fever virus epizootic in São Paulo, Brazil, 2016–2018. PLoS Pathog. 2020;16: 2016–2018. doi: 10.1371/JOURNAL.PPAT.1008699 32764827 PMC7437926

[25] Ministério da Saúde. Classificação de Risco dos Agentes Biológicos. In: Ministério da Saúde [Internet]. 2017 [cited 2 Sep 2019] pp. 1–50. Available from: http://bvsms.saude.gov.br/bvs/publicacoes/classificacao_risco_agentes_biologicos_3ed.pdf.

[26] Domingo C, Patel P, Yillah J, Weidmann M, Méndez JA, Nakouné ER, et al. Advanced yellow fever virus genome detection in point-of-care facilities and reference laboratories. J Clin Microbiol. 2012;50: 4054–4060. doi: 10.1128/JCM.01799-12 23052311 PMC3503008

[27] Avelino-Silva VI, Leal FE, Sabino EC, Nishiya AS, Da Silva Freire M, Blumm F, et al. Yellow fever vaccine viremia following ablative BM suppression in AML. Bone Marrow Transplant. Nature Publishing Group; 2013;48: 1008–1009. doi: 10.1038/bmt.2012.277 23334273

[28] Pereira RH, Gonçalves CN. geobr: Loads Shapefiles of Official Spatial Data Sets of Brazil. GitHub repository; 2019.

[29] Cunha M dos P, Duarte-Neto AN, Pour SZ, Hajjar LA, Frassetto FP, Dolhnikoff M, et al. Systemic dengue infection associated with a new dengue virus type 2 introduction in Brazil–a case report. BMC Infect Dis. BioMed Central Ltd; 2021;21: 1–6. doi: 10.1186/S12879-021-05959-2/FIGURES/4 33794785 PMC8015031

[30] Bolger AM, Lohse M, Usadel B. Trimmomatic: A flexible trimmer for Illumina sequence data. Bioinformatics. 2014;30: 2114–2120. doi: 10.1093/bioinformatics/btu170 24695404 PMC4103590

[31] Langmead B, Salzberg SL. Fast gapped-read alignment with Bowtie 2. Nat Methods. 2012;9: 357–359. doi: 10.1038/nmeth.1923 22388286 PMC3322381

[32] Okonechnikov K, Golosova O, Fursov M, Varlamov A, Vaskin Y, Efremov I, et al. Unipro UGENE: A unified bioinformatics toolkit. Bioinformatics. 2012;28: 1166–1167. doi: 10.1093/bioinformatics/bts091 22368248

[33] Larkin MA, Blackshields G, Brown NP, Chenna R, Mcgettigan PA, McWilliam H, et al. Clustal W and Clustal X version 2.0. Bioinformatics. 2007;23: 2947–2948. doi: 10.1093/bioinformatics/btm404 17846036

[34] Martin DP, Murrell B, Golden M, Khoosal A, Muhire B. RDP4: Detection and analysis of recombination patterns in virus genomes. Virus Evol. 2015;1: 1–5. doi: 10.1093/ve/vev003 27774277 PMC5014473

[35] Larsson A. AliView: a fast and lightweight alignment viewer and editor for large datasets. Bioinformatics. 2014;30: 3276–3278. doi: 10.1093/bioinformatics/btu531 25095880 PMC4221126

[36] Nguyen L-T, Schmidt HA, Haeseler A von, Minh BQ. IQ-TREE: A Fast and Effective Stochastic Algorithm for Estimating Maximum-Likelihood Phylogenies. Mol Biol Evol. 2015;32: 268–274. doi: 10.1093/molbev/msu300 25371430 PMC4271533

[37] Rambaut A. FigTree Version 1.4.4. 2018.

[38] Rambaut A, Lam TT, Max Carvalho L, Pybus OG. Exploring the temporal structure of heterochronous sequences using TempEst (formerly Path-O-Gen). Virus Evol. 2016;2: vew007. doi: 10.1093/ve/vew007 27774300 PMC4989882

[39] Suchard MA, Lemey P, Baele G, Ayres DL, Drummond AJ, Rambaut A. Bayesian phylogenetic and phylodynamic data integration using BEAST 1.10. Virus Evol. 2018;4: 1–5. doi: 10.1093/ve/vey016 29942656 PMC6007674

[40] Darriba D, Taboada GL, Doallo R, Posada D. JModelTest 2: More models, new heuristics and parallel computing. Nat Methods. Nature Publishing Group; 2012;9: 772. doi: 10.1038/nmeth.2109 22847109 PMC4594756

[41] Mir D, Delatorre E, Bonaldo M, Lourenço-De-Oliveira R, Vicente AC, Bello G. Phylodynamics of Yellow Fever Virus in the Americas: New insights into the origin of the 2017 Brazilian outbreak. Sci Rep. 2017;7: 1–9. doi: 10.1038/s41598-017-07873-7 28785067 PMC5547128

[42] Gill MS, Lemey P, Faria NR, Rambaut A, Shapiro B, Suchard MA. Improving bayesian population dynamics inference: A coalescent-based model for multiple loci. Mol Biol Evol. 2013;30: 713–724. doi: 10.1093/molbev/mss265 23180580 PMC3563973

[43] Minin VN, Bloomquist EW, Suchard MA. Smooth skyride through a rough skyline: Bayesian coalescent-based inference of population dynamics. Mol Biol Evol. 2008;25: 1459–1471. doi: 10.1093/molbev/msn090 18408232 PMC3302198

[44] Rambaut A, Drummond AJ. Tracer v1.4. Available from http://beast.bio.ed.ac.uk/Tracer. Available from http://beast.bio.ed.ac.uk/Tracer. 2007.

[45] Baele G, Li WLS, Drummond AJ, Suchard MA, Lemey P. Accurate model selection of relaxed molecular clocks in Bayesian phylogenetics. Mol Biol Evol. 2013;30: 239–243. doi: 10.1093/molbev/mss243 23090976 PMC3548314

[46] Baele G, Lemey P, Bedford T, Rambaut A, Suchard MA, Alekseyenko AV. Improving the accuracy of demographic and molecular clock model comparison while accommodating phylogenetic uncertainty. Mol Biol Evol. 2012;29: 2157–2167. doi: 10.1093/molbev/mss084 22403239 PMC3424409

[47] Baele G, Lemey P, Suchard MA. Genealogical Working Distributions for Bayesian Model Testing with Phylogenetic Uncertainty. Syst Biol. 2016;65: 250–264. doi: 10.1093/sysbio/syv083 26526428 PMC5009437

[48] Bielejec F, Baele G, Vrancken B, Suchard MA, Rambaut A, Lemey P. SpreaD3: Interactive Visualization of Spatiotemporal History and Trait Evolutionary Processes. Mol Biol Evol. 2016;33: 2167–2169. doi: 10.1093/molbev/msw082 27189542 PMC6398721

[49] Delatorre E, Abreu FVS, Ribeiro IP, Gómez MM, Santos AAC, Ferreira-De-Brito A, et al. Distinct YFV Lineages Co-circulated in the Central-Western and Southeastern Brazilian Regions from 2015 to 2018. Front Microbiol. 2019;10: 1–12. doi: 10.3389/fmicb.2019.01079 31178835 PMC6543907

[50] Possas C, Lourenço-de-Oliveira R, Tauil PL, Pinheiro F de P, Pissinatti A, Cunha RV da, et al. Yellow fever outbreak in Brazil: the puzzle of rapid viral spread and challenges for immunisation. Mem Inst Oswaldo Cruz. 2018;113: e180278. doi: 10.1590/0074-02760180278 30427974 PMC6135548

[51] CDC. Yellow Fever [Internet]. [cited 22 Jun 2022]. Available from: https://www.cdc.gov/yellowfever/index.html.

[52] Rudolph KE, Lessler J, Moloney RM, Kmush B, Cummings DAT. Incubation Periods of Mosquito-Borne Viral Infections: A Systematic Review. Am J Trop Med Hyg. 2014;90: 882–891. doi: 10.4269/ajtmh.13-0403 24639305 PMC4015582

[53] Casadio L, Nastri AC, Malta FM, Araujo J, Silva JB, Salomao J, et al. Late-Onset Relapsing Hepatitis Associated with Yellow Fever. new engl J Med. 2020; 2059–2061. doi: 10.1056/NEJMc1913036 32433844

[54] Ferreira MS, Soares P, Júnior B, Cerqueira VD, Riet G, Rivero C, et al. Experimental yellow fever virus infection in the squirrel monkey (Saimiri spp.) I: gross anatomical and histopathological findings in organs at necropsy. Mem Inst Oswaldo Cruz. 2020;115: 1–8. doi: 10.1590/0074-02760190501 33174908 PMC7651848

[55] Costa ZGA, Romano APM, Elkhoury ANM, Flannery B. Evolução histórica da vigilância epidemiológica e do controle da febre amarela no Brasil. Rev Pan-Amazônica Saúde. 2011;2: 11–26. doi: 10.5123/s2176-62232011000100002

[56] Fioravanti CH. O Combate à febre amarela no Estado de São Paulo: História, Desafios e Inovações. 2018.

[57] Hamrick PN, Aldighieri S, Machado G, Leonel DG, Vilca LM, Uriona S, et al. Geographic patterns and environmental factors associated with human yellow fever presence in the Americas. PLoS Negl Trop Dis. 2017;11: 1–27. doi: 10.1371/journal.pntd.0005897 28886023 PMC5607216

[58] Di Couto-Lima, Madec Y Bersot MI, Campos SS Motta MDA, Dos Santos FB, et al. Potential risk of re-emergence of urban transmission of Yellow Fever virus in Brazil facilitated by competent Aedes populations. Sci Reports 2017 71. Nature Publishing Group; 2017;7: 1–12. doi: 10.1038/s41598-017-05186-3 28687779 PMC5501812

[59] Giovanetti M, Mendonça MCL, Fonseca V, Mares-Guia MA, Fabri A, Xavier J, et al. Yellow Fever Virus Reemergence and Spread in Southeast Brazil, 2016–2019. J Virol. 2020;94: e01623–19.

[60] Secretaria de Estado da Saúde—Santa Catarina. Aumento do número de epizootias em Primatas Não Humanos (PNH) no ano de 2020. Nota Alerta 001/2020/DIVE/SUV/SES. 2020; 1–2.

[61] Secretaria da Saúde—Paraná. Monitoramento da Situação Epidemiológica da Febre Amarela no Paraná. Inf epidemiológico n° 019/2020. 2020; 1–7.

[62] Andrade M de S, Campos FS, Campos AAS, Abreu FVS, Melo FL, Sevá A da P, et al. Real-Time Genomic Surveillance during the 2021 Re-Emergence of the Yellow Fever Virus in Rio Grande do Sul State, Brazil. Viruses 2021, Vol 13, Page 1976. Multidisciplinary Digital Publishing Institute; 2021;13: 1976. doi: 10.3390/v13101976 34696408 PMC8539658

