# Supplementary material for: Phylogeographic patterns of the yellow fever virus around the metropolitan region of São Paulo, Brazil, 2016–2019
Source: PLoS Negl Trop Dis. 2022 Sep 23;16(9):e0010705. doi: 10.1371/journal.pntd.0010705 (PMC9506654; doi:10.1371/journal.pntd.0010705)
Supplement: S1 Text — Fig A in S1 Text. Combined coverage (normalized by the sample average) along the two sequenced Yellow Fever virus (YFV) genomes generated in this study. The genomic position reflects the genomic organization of the YFV, which is organized as follows: a single polyprotein cleaved into three structural, including capsid (C), membrane (M), and envelope (E), and seven non-structural (NS) proteins named NS1, NS2A, NS2B, NS3, NS4A, NS4B, and NS5. The polyprotein is flanked by the 5’ and 3’ ends, which are non-coding. Table A in S1 Text. Complete YFV polyprotein sequences used in the phylogenetic analysis (dataset-1) (n = 264). Table B in S1 Text. Complete YFV polyprotein sequences used in the phylogeographic analysis (dataset-2) (n = 91). Table C in S1 Text. Model comparison of the relaxed molecular clock and demographic growth models through path sampling (PS) and stepping stone (SS) methods. Bold numbers indicate the best fitting model. Table D in S1 Text. Comparison among continuous diffusion models for Brazilian sequences. (DOCX) [file pntd.0010705.s001.docx]

Supporting information for:

**Phylogeographic Patterns of the Yellow Fever virus around the Metropolitan Region of São Paulo, Brazil, 2016-2019**

Marielton dos Passos Cunha ^#^, Amaro Nunes Duarte-Neto, Shahab Zaki Pour, Bárbara Brito de Souza Pereira, Yeh-Li Ho, Beatriz Perondi, Jaques Sztajnbok, Venancio Avancini Ferreira Alves, Luiz Fernando Ferraz da Silva, Marisa Dolhnikoff, Paulo Hilário Nascimento Saldiva, Paolo Marinho de Andrade Zanotto

# **Corresponding author**: marieltondospassos@gmail.com (MPC)

**Contents:**

- Fig A
- Table A
- Table B
- Table C
- Table D


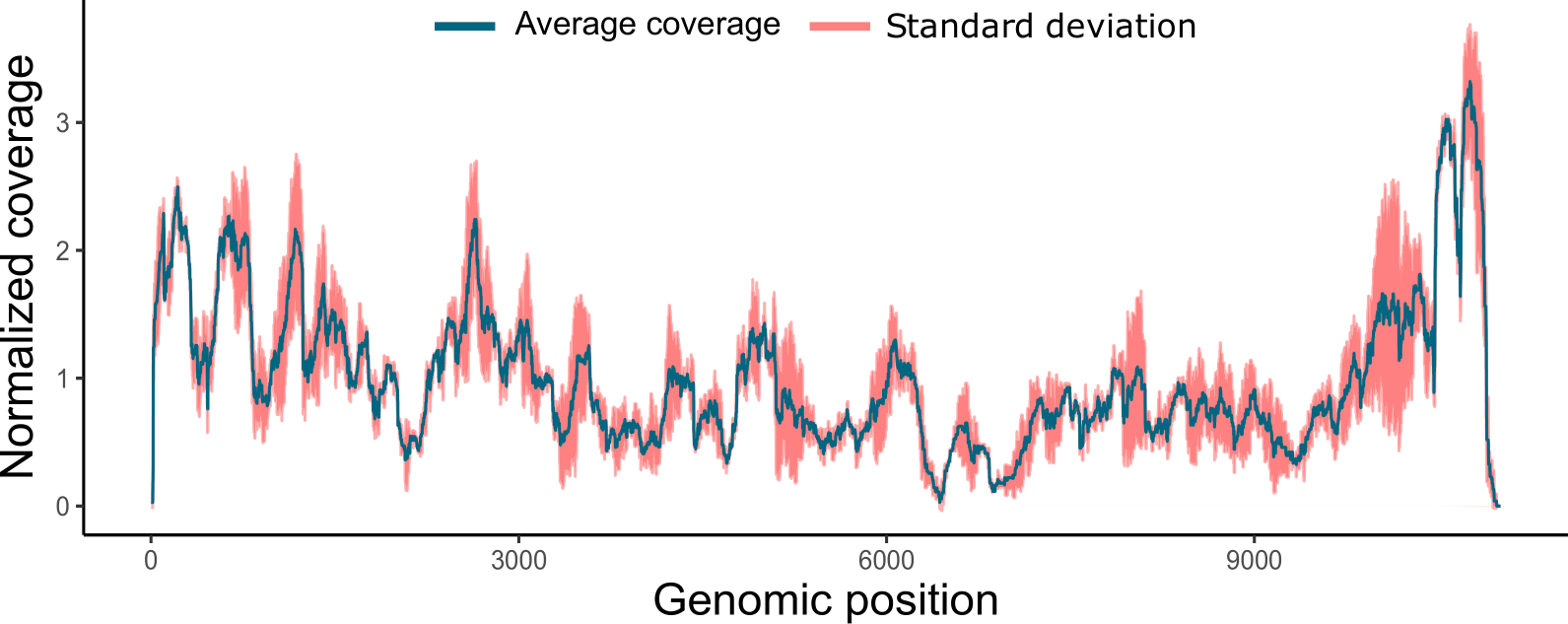
**Fig A**. Combined coverage (normalized by the sample average) along the two sequenced Yellow Fever virus (YFV) genomes generated in this study. The genomic position reflects the genomic organization of the YFV, which is organized as follows: a single polyprotein cleaved into three structural, including capsid (C), membrane (M), and envelope (E), and seven non-structural (NS) proteins named NS1, NS2A, NS2B, NS3, NS4A, NS4B, and NS5. The polyprotein is flanked by the 5' and 3' ends, which are non-coding.

**Table A**. Complete YFV polyprotein sequences used in the phylogenetic analysis (dataset-1) (n = 264).

| **GenBank number** | **Yellow Fever virus genotype** |
| --- | --- |
| MW308134.1 | South American I |
| MW308135.1 | South American I |
| MK583147.1 | South American I |
| MK333798.1 | South American I |
| MK583151.1 | South American I |
| MK583155.1 | South American I |
| MK583149.1 | South American I |
| MK583167.1 | South American I |
| MK583166.1 | South American I |
| MK583163.1 | South American I |
| MK583153.1 | South American I |
| MK583158.1 | South American I |
| MK583148.1 | South American I |
| MK333799.1 | South American I |
| MK583165.1 | South American I |
| MK583172.1 | South American I |
| MK583160.1 | South American I |
| MK583176.1 | South American I |
| MK583162.1 | South American I |
| MK583164.1 | South American I |
| MK583156.1 | South American I |
| MK583181.1 | South American I |
| MK583161.1 | South American I |
| MK583177.1 | South American I |
| MK583159.1 | South American I |
| MK583175.1 | South American I |
| MK583170.1 | South American I |
| MK583182.1 | South American I |
| MK583179.1 | South American I |
| MK583174.1 | South American I |
| MK583171.1 | South American I |
| MK583157.1 | South American I |
| MK583152.1 | South American I |
| MK583180.1 | South American I |
| MK583150.1 | South American I |
| MK583178.1 | South American I |
| MK583173.1 | South American I |
| MK760666.1 | South American I |
| MK583169.1 | South American I |
| MK333804.1 | South American I |
| MK728873.1 | South American I |
| MF370532.1 | South American I |
| MF370538.1 | South American I |
| MF370534.1 | South American I |
| MF423375.2 | South American I |
| MF423373.2 | South American I |
| MF423378.2 | South American I |
| MK333802.1 | South American I |
| KY885001.2 | South American I |
| MF423374.2 | South American I |
| MF370548.1 | South American I |
| MF370537.1 | South American I |
| MK333800.1 | South American I |
| MF538786.2 | South American I |
| MF434851.2 | South American I |
| MF370531.1 | South American I |
| MF370547.1 | South American I |
| MK583168.1 | South American I |
| MK333807.1 | South American I |
| MK333801.1 | South American I |
| MF370536.1 | South American I |
| MK760662.1 | South American I |
| MK333809.1 | South American I |
| MF538782.2 | South American I |
| MK333808.1 | South American I |
| MF538784.2 | South American I |
| MK333806.1 | South American I |
| MF423376.2 | South American I |
| MK760660.1 | South American I |
| MF538783.2 | South American I |
| MF465805.1 | South American I |
| MK760661.1 | South American I |
| MK583154.1 | South American I |
| MK760663.1 | South American I |
| MK760664.1 | South American I |
| MK533792.1 | South American I |
| MF370535.1 | South American I |
| MF370530.1 | South American I |
| MF370533.1 | South American I |
| MK760665.1 | South American I |
| MF370546.1 | South American I |
| MF538785.2 | South American I |
| MF370544.1 | South American I |
| MF370540.1 | South American I |
| MF370549.1 | South American I |
| KY861728.1 | South American I |
| MF370541.1 | South American I |
| MK333803.1 | South American I |
| MF370543.1 | South American I |
| JF912190.1 | South American I |
| MF370539.1 | South American I |
| MK333805.1 | South American I |
| MK089775.1 | South American I |
| MF370542.1 | South American I |
| MF170971.1 | South American I |
| JF912187.1 | South American I |
| MH018064.1 | South American I |
| MH018066.1 | South American I |
| MH193173.1 | South American I |
| MG969501.1 | South American I |
| MH018067.1 | South American I |
| MH030085.1 | South American I |
| MH030050.1 | South American I |
| MF170973.1 | South American I |
| MF170972.1 | South American I |
| MF170977.1 | South American I |
| MH030052.1 | South American I |
| MF170976.1 | South American I |
| MN117917.1 | South American I |
| MH030051.1 | South American I |
| MF170979.1 | South American I |
| MF170975.1 | South American I |
| MH030053.1 | South American I |
| MH018065.1 | South American I |
| MF170981.1 | South American I |
| MH030075.1 | South American I |
| MT497525.1 | South American I |
| MT497522.1 | South American I |
| MH030076.1 | South American I |
| MH030084.1 | South American I |
| MH030062.1 | South American I |
| MF170978.1 | South American I |
| MF170974.1 | South American I |
| MH030056.1 | South American I |
| MH030055.1 | South American I |
| MH030069.1 | South American I |
| MH030065.1 | South American I |
| JF912189.1 | South American I |
| MH030059.1 | South American I |
| MH030049.1 | South American I |
| MH030083.1 | South American I |
| MH030067.1 | South American I |
| MH030064.1 | South American I |
| MF170970.1 | South American I |
| MF170969.1 | South American I |
| MH030073.1 | South American I |
| MH030082.1 | South American I |
| MH030054.1 | South American I |
| MT497521.1 | South American I |
| MH030086.1 | South American I |
| MH030061.1 | South American I |
| MH030072.1 | South American I |
| MH193174.1 | South American I |
| MH030077.1 | South American I |
| MH030060.1 | South American I |
| MH030070.1 | South American I |
| MH030080.1 | South American I |
| MH030078.1 | South American I |
| MH030071.1 | South American I |
| MH018082.1 | South American I |
| MH030079.1 | South American I |
| MH018096.1 | South American I |
| MH018080.1 | South American I |
| MH018079.1 | South American I |
| MF170980.1 | South American I |
| MH193175.1 | South American I |
| MH030066.1 | South American I |
| MH030074.1 | South American I |
| MH030068.1 | South American I |
| MH018092.1 | South American I |
| MH018090.1 | South American I |
| MH030063.1 | South American I |
| MH030057.1 | South American I |
| MH030058.1 | South American I |
| MF170968.1 | South American I |
| MH018078.1 | South American I |
| MH018095.1 | South American I |
| MH030081.1 | South American I |
| MH018076.1 | South American I |
| MH018093.1 | South American I |
| MH018083.1 | South American I |
| MH018091.1 | South American I |
| MH378284.1 | South American I |
| MH018084.1 | South American I |
| MK882607.1 | South American I |
| MK882604.1 | South American I |
| MH018089.1 | South American I |
| MN604285.1 | South American I |
| MH018088.1 | South American I |
| MH484430.1 | South American I |
| MH484427.1 | South American I |
| MH484429.1 | South American I |
| MK882603.1 | South American I |
| MK249065.1 | South American I |
| MH018099.1 | South American I |
| MF370545.1 | South American I |
| JF912188.1 | South American I |
| MH484434.1 | South American I |
| KM388817.1 | South American I |
| KM388816.1 | South American I |
| JF912180.1 | South American I |
| KM388814.1 | South American I |
| KM388818.1 | South American I |
| KM388815.1 | South American I |
| JF912185.1 | South American I |
| JF912182.1 | South American I |
| JF912186.1 | South American I |
| JF912184.1 | South American I |
| MH484426.1 | South American I |
| JF912179.1 | South American I |
| JF912183.1 | South American I |
| MH018101.1 | South American I |
| MH018100.1 | South American I |
| HM582851.1 | South American I |
| MF347613.1 | South American I |
| JF912181.1 | South American II |
| MF004382.1 | South American II |
| JX898869.1 | West African |
| KU978765.1 | West African |
| JX898868.1 | West African |
| JX898870.1 | West African |
| KU978764.1 | West African |
| KU978763.1 | West African |
| JX898874.1 | West African |
| JX898873.1 | West African |
| JX898875.1 | West African |
| JX898876.1 | West African |
| MK292067.1 | West African |
| MF405338.1 | West African |
| JX898871.1 | West African |
| JX898878.1 | West African |
| JX898880.1 | West African |
| JX898877.1 | West African |
| JX898872.1 | West African |
| MK457701.1 | West African |
| GQ379162.1 | West African |
| MN708496.1 | West African |
| GQ379163.1 | West African |
| MN708497.1 | West African |
| MN708495.1 | West African |
| MN708494.1 | West African |
| MN708492.1 | West African |
| MN708488.1 | West African |
| MN708490.1 | West African |
| MF289572.1 | West African |
| DQ118157.1 | West African |
| MN708489.1 | West African |
| MN708493.1 | West African |
| MN708491.1 | West African |
| MG922934.1 | West African |
| MN958078.1 | West African |
| JX949181.1 | West African |
| MF926243.1 | West African |
| MK060080.1 | West African |
| KX010994.1 | East African |
| KX268355.1 | East African |
| KY587416.1 | East African |
| KY495641.1 | East African |
| KU921608.1 | East African |
| KX027336.1 | East African |
| MG589641.1 | East African |
| KY873607.1 | East African |
| KX010995.1 | East African |
| JN620362.1 | East African |
| MH633686.1 | East African |
| MH633684.1 | East African |
| KX010996.1 | East African |
| MH633688.1 | East African |
| MH633685.1 | East African |
| MH633691.1 | East African |
| MH633692.1 | East African |
| MH633687.1 | East African |
| MH633689.1 | East African |
| MH633690.1 | East African |

**Table B**. Complete YFV polyprotein sequences used in the phylogeographic analysis (dataset-2) (n = 91).

| **GenBank number** | **Location** | **Date** |
| --- | --- | --- |
| MW308134.1 | Brazil - Caraguatatuba, SP | 26/10/2018 |
| MW308135.1 | Brazil - Iporanga, SP | 20/01/2019 |
| MK583147.1 | Brazil - Mairiporã, SP | 12/01/2018 |
| MH030085.1 | Brazil - Piracaia, SP | 15/11/2017 |
| MH030050.1 | Brazil - Louveira, SP | 16/08/2017 |
| MK333798.1 | Brazil - São Paulo, SP | 08/01/2018 |
| MK583151.1 | Brazil - Mairiporã, SP | 15/01/2018 |
| MK583155.1 | Brazil - Mairiporã, SP | 22/01/2018 |
| MK583149.1 | Brazil - São Paulo, SP | 16/01/2018 |
| MK583167.1 | Brazil - Guarulhos, SP | 11/02/2018 |
| MH030051.1 | Brazil - Louveira, SP | 15/09/2017 |
| MK583166.1 | Brazil - São Paulo, SP | 06/02/2018 |
| MK583163.1 | Brazil - São Paulo, SP | 02/02/2018 |
| MH030053.1 | Brazil - Itatiba, SP | 30/08/2017 |
| MK583153.1 | Brazil - Mairiporã, SP | 20/01/2018 |
| MK583158.1 | Brazil - Mairiporã, SP | 23/01/2018 |
| MH030075.1 | Brazil - Jarinu, SP | 15/10/2017 |
| MT497525.1 | Brazil - Ribeirão Preto, SP | 21/01/2017 |
| MT497522.1 | Brazil - Jaboticabal, SP | 15/11/2016 |
| MH030076.1 | Brazil - Jarinu, SP | 15/10/2017 |
| MH030084.1 | Brazil - Piracaia, SP | 15/11/2017 |
| MH030062.1 | Brazil - Itatiba, SP | 19/09/2017 |
| MK583148.1 | Brazil - Mairiporã, SP | 13/01/2018 |
| MK333799.1 | Brazil - São Paulo, SP | 08/01/2018 |
| MH030056.1 | Brazil - Jundiaí, SP | 12/09/2017 |
| MH030055.1 | Brazil - Jundiaí, SP | 11/09/2017 |
| MH030069.1 | Brazil - São Paulo, SP | 15/10/2017 |
| MH030065.1 | Brazil - Jundiaí, SP | 15/10/2017 |
| MK583165.1 | Brazil - Mairiporã, SP | 04/02/2018 |
| MK583172.1 | Brazil - Guarulhos, SP | 19/02/2018 |
| MH030049.1 | Brazil - Vinhedo, SP | 17/08/2017 |
| MK583160.1 | Brazil - Cotia, SP | 29/01/2018 |
| MH030067.1 | Brazil - Jundiaí, SP | 15/10/2017 |
| MH030064.1 | Brazil - Jundiaí, SP | 15/10/2017 |
| MK583176.1 | Brazil - Piedade, SP | 27/02/2018 |
| MK583162.1 | Brazil - São Paulo, SP | 31/01/2018 |
| MH030073.1 | Brazil - Mairiporã, SP | 18/10/2017 |
| MH030082.1 | Brazil - CampoLimpo, SP | 15/11/2017 |
| MH030054.1 | Brazil - Jundiaí, SP | 15/09/2017 |
| MT497521.1 | Brazil - Pindorama, SP | 13/10/2016 |
| MH030086.1 | Brazil - Piracaia, SP | 15/11/2017 |
| MH030061.1 | Brazil - Itatiba, SP | 21/09/2017 |
| MH030072.1 | Brazil - Jarinu, SP | 15/10/2017 |
| MH193174.1 | Brazil - Mairiporã, SP | 09/01/2018 |
| MH030077.1 | Brazil - Jarinu, SP | 15/10/2017 |
| MH030060.1 | Brazil - Itatiba, SP | 27/09/2017 |
| MH030070.1 | Brazil - Campo Limpo Paulista, SP | 19/10/2017 |
| MH030080.1 | Brazil - Nazaré Paulista, SP | 15/11/2017 |
| MH030078.1 | Brazil - Morungaba, SP | 15/10/2017 |
| MH030071.1 | Brazil - Campo Limpo Paulista, SP | 18/10/2017 |
| MK583164.1 | Brazil - Cotia, SP | 03/02/2018 |
| MH030079.1 | Brazil - Nazaré Paulista, SP | 15/11/2017 |
| MK583156.1 | Brazil - Atibaia, SP | 23/01/2018 |
| MK583181.1 | Brazil - São Lourenço da Serra, SP | 27/03/2018 |
| MK583161.1 | Brazil - Itaquaquecetuba, SP | 24/01/2018 |
| MK583177.1 | Brazil - Guarulhos, SP | 04/03/2018 |
| MH193175.1 | Brazil - São Paulo, SP | 01/02/2018 |
| MH030066.1 | Brazil - Jundiaí, SP | 15/10/2017 |
| MH030074.1 | Brazil - Jarinu, SP | 18/10/2017 |
| MH030052.1 | Brazil - Louveira, SP | 15/09/2017 |
| MH030068.1 | Brazil - Jundiaí, SP | 15/10/2017 |
| MK583159.1 | Brazil - Atibaia, SP | 24/01/2018 |
| MK583175.1 | Brazil - Itaquaquecetuba, SP | 27/02/2018 |
| MH030063.1 | Brazil - Itatiba, SP | 15/10/2017 |
| MH030057.1 | Brazil - Bragança Paulista, SP | 27/09/2017 |
| MH030058.1 | Brazil - Itatiba, SP | 26/09/2017 |
| MK583170.1 | Brazil - Guarulhos, SP | 18/02/2018 |
| MK583182.1 | Brazil - Guarulhos, SP | 14/03/2018 |
| MK583179.1 | Brazil - Ibiuna, SP | 11/03/2018 |
| MH030081.1 | Brazil - Campo Limpo, SP | 15/11/2017 |
| MK583174.1 | Brazil - Arujá, SP | 24/02/2018 |
| MK583171.1 | Brazil - Guarulhos, SP | 18/02/2018 |
| MK583157.1 | Brazil - Guarulhos, SP | 23/01/2018 |
| MK583152.1 | Brazil - Ibiúna, SP | 16/01/2017 |
| MK583180.1 | Brazil - Guarulhos, SP | 14/03/2018 |
| MK583150.1 | Brazil - Itaquaquecetuba, SP | 15/01/2018 |
| MK583178.1 | Brazil - Itariri, SP | 10/03/2018 |
| MK583173.1 | Brazil - Guarulhos, SP | 23/02/2018 |
| MK583169.1 | Brazil - Guarulhos, SP | 18/02/2018 |
| MH193173.1 | Brazil - Guarulhos, SP | 09/01/2018 |
| MH030059.1 | Brazil - Itatiba, SP | 21/09/2017 |
| MH030083.1 | Brazil - Campo Limpo, SP | 15/11/2017 |
| MH018064.1 | Brazil - Santa Rita de Caldas, MG | 15/02/2017 |
| MH018066.1 | Brazil - Ouro Fino, MG | 15/02/2017 |
| MH018067.1 | Brazil - Caldas, MG | 13/02/2017 |
| MH018065.1 | Brazil - Delfinópolis, MG | 14/02/2017 |
| MH018082.1 | Brazil - Claraval, MG | 20/02/2017 |
| MH484430.1 | Brazil - Sacramento, MG | 09/02/2017 |
| MF170971.1 | Brazil - São Roque de Minas, MG | 30/01/2017 |
| MK333804.1 | Brazil - Novo Brasil, GO | 17/08/2015 |
| MK728873.1 | Brazil - Amorinópolis, GO | 04/03/2017 |

**Table C**. Model comparison of the relaxed molecular clock and demographic growth models (dataset-2) through path sampling (PS) and stepping stone (SS) methods. Bold numbers indicate the best fitting model.

|  | **Relaxed molecular clock** | |
| --- | --- | --- |
| **Demographic growth model** | **PS** | **SS** |
| **Bayesian skyline plot** | **-16528.06** | **-16528.63** |
| Bayesian skyride plot | -16557.78 | -16558.25 |
| Bayesian skygrid model | -16530.12 | -16530.23 |

PS: Path sampling; SS: Stepping stone sampling.

**Table D**. Comparison among continuous diffusion models for Brazilian YFV sequences (dataset-2).

| **Continuous diffusion models** | **PS** | **SS** | **BEAST likelihood (stdev)** | **Coefficient of variation (95% HPD)** | **Correlation (95% HPD)** | **Dispersal rate (95% HPD)** |
| --- | --- | --- | --- | --- | --- | --- |
| BD | -16528.06 | -16528.63 | -16163.72 (11.9) | 1.23 (0.77, 1.73) | 0.07 (-0.139, 0.317) | 113.698 (78.99, 149.46) |
| Cauchy RRW model | -16485.70 | -16487.13 | -16065.21 (17.65) | 0.522 (0.145, 0.886) | 0.084 (-0.229, 0.379) | 130.354 (99.82, 161.08) |
| **Gamma RRW model** | **-16478.52** | **-16480.47** | **-16035.78 (18.13)** | **0.544 (0.208, 1.022** | **0.076 (-0.233, 0.349)** | **128.932 (98.85, 161.82)** |
| Lognormal RRW model | -16496.89 | -16497.30 | -16097.23 (16.59) | 0.603 (0.215, 0.975) | 0.063 (-0.241, 0.322) | 137.438 (106.11, 169.89) |

BD: Brownian diffusion process; RRWs: Relaxed random walks; PS: Path sampling; SS: Stepping stone.
